# Entropy of a Quasi-de Sitter Spacetime and the Role of Specific Heat

**DOI:** 10.3390/e28010043

**Published:** 2025-12-30

**Authors:** Orlando Luongo, Maryam Azizinia, Kuantay Boshkayev

**Affiliations:** 1 School of Science and Technology, University of Camerino, Via Madonna delle Carceri, 62032 Camerino, Italy; maryam.azizinia@unicam.it; 2 Osservatorio Astronomico di Brera, Istituto Nazionale di Astrofisica (INAF), 20121 Milano, Italy; 3 Istituto Nazionale di Fisica Nucleare (INFN), Sezione di Perugia, 06123 Perugia, Italy; 4 Faculty of Physics and Technology, Al-Farabi Kazakh National University, Almaty 050040, Kazakhstan; kuantay@mail.ru

**Keywords:** black holes, thermodynamics, de Sitter space

## Abstract

We investigate the thermodynamic properties of a generalized de Sitter-like configuration. This investigation proceeds in two essential steps: (1) first, we construct a spacetime whose energy–momentum tensor asymptotically reproduces quintessence while maintaining isotropic pressures, despite being fueled by a nonconstant energy–momentum tensor; (2) second, we define a finite domain of validity for the solution, within which an additional Cauchy horizon emerges. Afterwards, we analyze the thermodynamic behavior of this configuration and compare it with the standard de Sitter case. Our results indicate that the extra parameter introduced in the metric does not lead to a positive specific heat; this value remains negative, suggesting that the role of such a parameter is thermodynamically nonessential.

## 1. Introduction

Recent progress in observational astrophysics has significantly intensified the theoretical modeling of black holes [1,2]. Exploring black hole dynamics in the presence of external fields, such as dilatons [3,4,5,6], quintessence and dark energy [7,8,9,10,11], quasi-quintessence and generalized K-essence [12] or, more broadly, within dark energy-dominated environments [13], offers a promising avenue for elucidating the interplay between compact objects and cosmological large-scale structures [14,15,16].

Parallel theoretical developments have addressed the existence and the properties of nonsingular black hole solutions, often referred to as regular black holes [17,18,19,20]. These models, obtained as exact or approximate solutions of Einstein’s field equations, fundamentally alter the internal geometry of black holes, typically violating the energy conditions and making the curvature invariants regular at the core [21,22,23,24,25,26]. A highly relevant key feature of such solutions is that they may incorporate *effective vacuum energy cores*, as exemplified by the Hayward geometry [27] and its subsequent generalizations. In this respect, the regular spacetimes may be seen as hairy black holes, where the central region is characterized by a de Sitter-like core, smoothly transitioning to an asymptotically Schwarzschild spacetime at large radii, effectively replacing the classical singularity with a regular phase of constant positive curvature.

This appears consistent with the current understanding of cosmology, where the initial, naked, purely classical singularity may be mitigated by quantum effects, thereby avoiding *de facto* a classical Big Bang; such mitigation can be achieved by incorporating a vacuum energy contribution from the de Sitter metric at primordial times. Accordingly, de Sitter and anti-de Sitter spaces appear particularly intriguing in the context of framing gravitational situations. The great advantage of such solutions is that they can admit a correspondence with conformal field theories, as in the anti-de Sitter case, and they can exhibit a energy–momentum tensor that exhibits constant effective vacuum energy in the form of a unbounded cosmological constant. Moreover, the de Sitter phase finds applications in pure cosmology and serves as a prototypical example of a regular solution. Although they are extremely interesting, such solutions do not incorporate mass as possible gravitational generator of a compact object made by pure vacuum energy. Moreover, the energy–momentum tensor is purely constant. Quite surprisingly, the anti-de Sitter solution appears to also be unstable [28,29], and the corresponding thermodynamics of the de Sitter space show, in analogy to the Schwarzschild solution, a negative specific heat, which results in thermodynamic instability.

Motivated by the above considerations, we here explore a class of deformed de Sitter phases, limiting our attention to the case of a positive cosmological constant and thus assuming the existence of a horizon. Accordingly, we propose a generalization of de Sitter space that includes an additional term that extends the solution to a nontrivial energy–momentum tensor that asymptotically reproduces the effects of quintessence. Locally, and as the radial coordinate approaches inner values toward the core, the solution exhibits negative energy and the emergence of a Cauchy horizon, extending the pure de Sitter geometry to include a ring in which observers may propagate. The corresponding thermodynamic properties are thus explored and compared with those of the de Sitter space. The entropy, temperature and specific heat are thus computed, and the deviations from the case of a pure vacuum energy case are found. We emphasize that the solution appears to depend on the extra term, although we see that, after manipulation, it is possible to demonstrate that the extra term has no effect. Hence, the thermodynamics of the solution so obtained remain fully unaltered, suggesting that the capacity to transport vacuum energy is the principal characteristic that gives the system its defining thermodynamic properties. Moreover, the thermodynamic instability associated with the specific heat persists, like due to the symmetry of the solution and the fact that we are limiting ourselves to general relativity. Possible approaches to resolving these issues and to extending our solutions are explored in the final section of this paper.

The paper is organized as follows. In Section 2, we introduce the main features of de Sitter space. In Section 3, the class of deformed de Sitter spacetimes is summarized. In Section 4, the corresponding thermodynamics are analyzed, and in Section 5, we discuss our solutions, along with the conclusions and future perspectives of this work.

## 2. Summary of de Sitter Space Thermodynamics

We work within Einstein’s gravity with a positive cosmological constant Λ>0, adopting the metric signature (−,+,+,+) and starting from Einstein’s field equations $G_{\mu \nu }+\Lambda \;g_{\mu \nu }=8\pi G\;T_{\mu \nu }$, recalling that the de Sitter solution satisfies

(1) $$\begin{array}{cc} \; & R_{\mu \nu }=\Lambda \;g_{\mu \nu }, \end{array}$$

(2) $$\begin{array}{cc} \; & R=4\Lambda , \end{array}$$

(3) $$\begin{array}{cc} \; & R_{\mu \nu \rho \sigma }=\frac{\Lambda }{3}\left(g_{\mu \rho }g_{\nu \sigma }-g_{\mu \sigma }g_{\nu \rho }\right). \end{array}$$

Employing the *static patch*, as seen by a timelike observer and in analogy to cosmology, we adopt the following nomenclature

(4) $$H\equiv \sqrt{\frac{\Lambda }{3}},$$

writing up the static-patch line element as follows:

(5) $$ds^{2}=-\left(1-H^{2}r^{2}\right)\;dt^{2}+\frac{dr^{2}}{1-H^{2}r^{2}}+r^{2}\;d\Omega_{2}^{2},$$

with $d\Omega_{2}^{2}$ the unit two-sphere metric.

The static Killing field is $\chi =\partial_{t}$. The cosmological horizon is the Killing horizon where $\chi^{2}=g_{tt}=-\left(1-H^{2}r^{2}\right)$ vanishes, as follows:

(6) $$1-H^{2}r^{2}=0\;\Rightarrow \;r=r_{h}=H^{-1},$$

Thus, the corresponding area associated with the horizon reads

(7) $$A_{c}=4\pi r_{h}^{2}=4\pi H^{-2}=\frac{12\pi }{\Lambda }.$$

From the above definitions, it is easy to argue the corresponding thermodynamics, split into the computations of entropy, temperature and, accordingly, specific heats.

### 2.1. Entropy and Surface Gravity

To define the temperature, a Killing horizon generated by χ has *surface gravity* κ defined by

(8) $$\chi^{\nu }\nabla_{\nu }\chi^{\mu }=\kappa \;\chi^{\mu },$$

on the horizon that equivalently reads

(9) $$\kappa^{2}=-\frac{1}{2}\left(\nabla_{\mu }\chi_{\nu }\right)\left(\nabla^{\mu }\chi^{\nu }\right){|}_{r=r_{h}}.$$

Thus, since our de Sitter metric appears as a static, spherically symmetric spacetime of the form $ds^{2}=-f\left(r\right)\;dt^{2}+\frac{dr^{2}}{f(r)}+r^{2}d\Omega_{2}^{2}$, identifying $f=g=1-\frac{1}{3}\Lambda r^{2}$, having a simple root $f(r_{h})=0$, one immediately finds

(10) $$\kappa =\frac{|f^{\prime }\left(r_{h}\right)|}{2},$$

with $f^{\prime }\left(r\right)=-2H^{2}r$, $f^{\prime }\left(r_{h}\right)=-2H$ giving rise to κ=H.

The associated Hawking temperature is therefore

(11) $$T=\frac{\kappa }{2\pi }=\frac{\hbar H}{2\pi },$$

where the Boltzmann constant is $k_{B}=1$ in our unit convention.

Accordingly, we have a Hawking temperature of the form

(12) $$T=\frac{1}{2\pi }\sqrt{\frac{\Lambda }{3}}.$$

At this stage, the entropy of any Killing horizon equals one quarter of its area,

(13) $$S=\frac{A}{4},$$

leading, for de Sitter, to

(14) $$S=\frac{A}{4}=\frac{\pi }{H^{2}}=\frac{3\pi }{\Lambda }.$$

#### Euclidean Derivation for the Temperature

The same result follows from imposing regularity of the Euclidean metric via a Wick rotation, namely t→−iτ, obtaining $ds_{E}^{2}=f\left(r\right)\;d\tau^{2}+\frac{dr^{2}}{f(r)}+r^{2}d\Omega_{2}^{2}$. Near the horizon, this metric can be approximated as follows:

(15) $$f\left(r\right)\approx f^{\prime }\left(r_{h}\right)\left(r-r_{h}\right)=-2H\left(r-r_{h}\right).$$

An appropriate coordinate change can thus be used to recast the de Sitter metric into a Rindler spacetime. So, defining ρ via

(16) $$d\rho^{2}=\frac{dr^{2}}{f(r)}\approx \frac{dr^{2}}{-2H(r-r_{h})},$$

and

(17) $$f\left(r\right)\approx 2H\left(r_{h}-r\right)\approx H^{2}\rho^{2},$$

we conclude that the metric near the horizon becomes

(18) $$ds_{E}^{2}\approx \rho^{2}H^{2}\;d\tau^{2}+d\rho^{2}+r_{h}^{2}d\Omega_{2}^{2}.$$

Hence, the (ρ,τ) plane is regular only if τ has period

(19) $$\beta =\frac{2\pi }{H},$$

which yields

(20) $$T=\frac{1}{\beta }=\frac{H}{2\pi },$$

in agreement with the surface gravity calculation.

### 2.2. Specific Heat of de Sitter Space

The thermodynamic behavior of de Sitter space can be further analyzed by studying the *specific heat* associated with the cosmological horizon. In semiclassical gravity, one can treat the horizon as a thermodynamic system characterized by entropy *S*, temperature *T*, and an effective energy *E*.

The *specific heat* is defined as

(21) $$C\equiv \frac{\partial E}{\partial T}=T\frac{\partial S}{\partial T},$$

where we express dE=TdS as a function of *T* by eliminating *H*. From T=H/(2π), we have

(22) $$H=2\pi T,\;r_{h}=\frac{1}{H}=\frac{1}{2\pi T},$$

indicating that

(23) $$S=\frac{1}{4\pi T^{2}}$$

This result is consistent with the fact that S>0.

Substituting into the expression for the specific heat, we obtain

(24) $$C=-\frac{1}{2\pi T^{2}}.$$

The result indicates, this time, that C≡−2S.

One arrives at the same result, noticing that $C\equiv T\frac{\partial S}{\partial r_{h}}{\left(\frac{\partial T}{\partial r_{h}}\right)}^{-1}$.

The negative sign of *C* shows that the de Sitter cosmological horizon, like the Schwarzschild black hole horizon, has a *negative specific heat*. This indicates that the thermodynamic system is *thermodynamically unstable in a canonical ensemble*, as an increase in energy reduces the temperature.

In terms of the cosmological constant, Λ, we obtain

(25) $$T=\frac{1}{2\pi r_{h}},\;C=-\frac{1}{2\pi }\cdot {\left(2\pi r_{h}\right)}^{2}=-2\pi r_{h}^{2}=-\frac{6\pi }{\Lambda }.$$

This result emphasizes the similarity between de Sitter horizons and black hole horizons: both exhibit thermodynamic instability in canonical ensembles, as expected for self-gravitating systems.

One may ask whether this result is general, or, in other words, whether the above occurrence is a direct consequence of the spherical symmetry alone. In addition, one may wonder under which circumstances a spherically symmetric object could exist without a central mass, *M*, supported only by a cosmological constant. The presence of mass, in fact, is essential for characterizing known compact objects, while de Sitter or generalized de Sitter space contain only Λ, without an associated central mass.

In general, recent observations have not revealed objects that have exact spherical symmetry, instead indicating the presence of quadrupole moments or angular momentum [30]. The *Kerr hypothesis* states that compact objects are described by either Kerr or Schwarzschild metrics. However, thermodynamic instability appears to be a symmetry-related effect and suggests, that at least within general relativity, compact objects endowed with exact spherical symmetry may not truly exist in nature. On the other hand, observations seem to indicate that astrophysical compact objects are rotating, and therefore may point toward excluding those solutions that exhibit negative specific heats. Hence, although spherical symmetry remains essential from a theoretical viewpoint, more studies are needed in light of the possible thermodynamic instability that may preclude the existence of purely spherically symmetric compact configurations.

Below, we therefore deform the de Sitter space, computing the corresponding thermodynamics.

## 3. Generalization of de Sitter Space

Generalized de Sitter space or, more briefly, quasi-de Sitter space, can be argued following the argument raised in Refs. [31,32].

The corresponding metrics can be obtained in two different ways, as reported below.

- Extending de Sitter space. This procedure requires finding *the most general metric that emulates de Sitter, providing isotropic pressures that are not constant, as well as an energy density that is a function of the radial coordinate*.
- Requiring a finite region with an emergent Cauchy horizon. In this way, it is possible to construct a region analogous to the de Sitter space, in which an observer placed in the center of the reference frame encounters a Cauchy horizon, which does not exist in pure de Sitter space.

For the first approach, we can consider a spherically symmetric spacetime [33]

(26) $$ds^{2}=-f\left(r\right)\;dt^{2}+\frac{1}{g(r)}\;dr^{2}+h\left(r\right)\;d\Omega^{2},$$

whose stationary region, where the Killing field $\partial_{t}$ is timelike, corresponds to the domain in which all three functions remain positive. In this coordinate chart (t,r,θ,φ), the energy–momentum tensor of an anisotropic fluid takes the diagonal form

(27) $$\left[T_{\nu }^{\mu }\right]=\left[\begin{array}{cccc} -\rho & 0 & 0 & 0\\ 0 & P_{r} & 0 & 0\\ 0 & 0 & P_{t} & 0\\ 0 & 0 & 0 & P_{t} \end{array}\right],$$

where ρ denotes the energy density, while $P_{r}$ and $P_{t}$ represent the radial and tangential pressures, respectively.

Imposing spherical symmetry, one may always redefine the radial coordinate such that $h\left(r\right)=r^{2}$, which is exactly the case that we consider below. The resulting general metric describes the spherically symmetric solution of an anisotropic fluid, in which we intend to impose isotropic pressure and include a cosmological constant as part of the energy–momentum tensor, although it is quite different from the de Sitter case.

The sign of Λ plays a crucial role in determining whether the spacetime is asymptotically de Sitter (Λ>0) or anti-de Sitter (Λ<0), with well-known implications for quantum field theory, holography, and the AdS/CFT correspondence [34].

This correspondence asserts a duality between a (d+1)–dimensional gravitational theory defined on an asymptotically anti-de Sitter (AdS) spacetime and a *d*–dimensional conformal field theory (CFT) living on its boundary.

More precisely, the duality asserts an exact equivalence between the partition functions $Z_{\mathrm{grav}}\left[\phi_{0}\right]\;=\;Z_{\mathrm{CFT}}\left[J\right]$, where $\phi_{0}$ denotes the boundary value of a bulk field ϕ, acting as a source *J* for the corresponding operator O in the CFT. Although exploring this is far beyond the scope of the present work, we leave open the possibility of extending the correspondence to a space that resembles the AdS, such as the one that we report below. In other words, our deformed AdS mass may provide a framework for generalizing the correspondence, further motivating the exploration of ways to extend standard AdS and dS frameworks via additional metrics that look similar to the original formulation.

Black hole thermodynamics in AdS space, for instance, exhibit the Hawking–Page transition.

In our framework, we focus exclusively on positive Λ to ensure the existence of an horizon and, accordingly, a thermodynamic framework, and we require that *corresponding metric asymptotically resembles quintessence*.

Accordingly, for quintessence, additional restrictions over the equation of state are needed. Specifically, defining $w\equiv \frac{P}{\rho }$ and having $P_{r}=P_{t}=P$, we require that asymptotically −1<w<0.

Motivated by this framework, it is natural to consider the following:

- a class of regular, spherically symmetric spacetimes allowing for a vacuum energy contribution capable of inducing a de Sitter phase with a (not necessarily constant) effective energy density Λ;
- an equation-of-state parameter *w* approaching a constant asymptotic value, consistent with experimental constraints [35,36], thereby enabling the existence of a dark energy component that may violate the WEC.

Incorporating a deformation of the standard de Sitter space, we can write

(28) $$ds^{2}=-\left(1-\frac{\Lambda }{3}\;r^{2+\epsilon }\right)dt^{2}+\frac{1}{g(r)}dr^{2}+r^{2}d\Omega^{2}\;,$$

where deviations from the pure de Sitter geometry are encoded in ϵ and in the requirement to have isotropic but nonconstant pressures. Specifically, g(r) is chosen to be regular at r=0 and to grow as $g\left(r\right)∼r^{2}$ in the limit r→∞, thus restoring a de Sitter asymptotic regime.

Thus, from the Einstein field equations, we end up with

(29) g(r)=f(r)F12−2ϵ+2,−ϵϵ+4;ϵϵ+2;Λ6(ϵ+4)rϵ+2+k0r21−Λ6(ϵ+4)rϵ+2ϵϵ+4+1,

where F12 is the analytical continuation of the hypergeometric function and $k_{0}$ a further integration constant, whose meaning will be clarified later in the text. The above expression turns out to be particularly interesting in the case ϵ=0 and $k_{0}\neq -\frac{2}{3}\Lambda$, giving rise to

(30) $$ds^{2}=-\left(1-\frac{\Lambda }{3}r^{2}\right)dt^{2}+\frac{\left(1-\frac{2}{3}\Lambda r^{2}\right)dr^{2}}{\left(1-\frac{\Lambda }{3}r^{2}\right)\left(1+k_{0}r^{2}\right)}+r^{2}d\Omega_{2}^{2},$$

where the significance of the integration constant $k_{0}$ is not known *a priori*. Indeed, in the original formulation of the metric, see Ref. [31], no definitive conclusions were drawn concerning this constant. There, it was argued that, when ϵ→0, de Sitter solution is not recovered *not because of the presence of $k_{0}$* but rather because $k_{0}$ is *a priori* unbounded and may significantly depart from the case $k_{0}=-\frac{2}{3}\Lambda$; this is in fact required to recover de Sitter space. Thus, in this work, we aim at finding a possible connection between this further constant, $k_{0}$, and the underlying thermodynamics of our extended de Sitter space.

Concerning the second approach, the above metric can be derived alternatively from the previously obtained solution, constructed by starting from a spherically symmetric, singular seed metric subjected to an additive deformation, as proposed in Ref. [37].

We assume that the metric components can be expressed via a δ–expansion, where δ parameterizes deviations from the vacuum Schwarzschild–de Sitter solution. Thus, ensuring $\chi \left(r\right)=1-\frac{2M}{r}-\frac{\Lambda }{3}r^{2}$, we find explicitly $f\left(r\right)=\chi \left(r\right)+\delta f_{1}\left(r\right)+O\left(\delta^{2}\right)$ and $g\left(r\right)=\chi \left(r\right)+\delta g_{1}\left(r\right)+O\left(\delta^{2}\right)$.

Accordingly, the same class of solutions previously analyzed can be recovered by starting from a spherically symmetric singular background and introducing an additive deformation, following the approach of Ref. [37]. To this end, we assume that the metric functions admit a δ–expansion, where the parameter δ quantifies the deviation from the vacuum solution with a cosmological constant, i.e., from the Schwarzschild-de Sitter spacetime. Accordingly, we write

(31) $$\begin{array}{cc} f(r) & =\chi \left(r\right)+\delta \;f_{1}\left(r\right)+\ldots , \end{array}$$

(32) $$\begin{array}{cc} g(r) & =\chi \left(r\right)+\delta \;g_{1}\left(r\right)+\ldots , \end{array}$$

with $\chi \left(r\right)=1-\frac{2M}{r}-\frac{\Lambda }{3}r^{2}$. The isotropy condition can be considerably simplified by introducing the additional ansatz

(33) $$f_{1}\left(r\right)=\chi \left(r\right)\;\alpha \left(r\right),\;g_{1}\left(r\right)=\chi \left(r\right)\;\beta \left(r\right).$$

Focusing on the *exact linear solutions*, obtained by setting α(r)=0, one finds

(34) $$f\left(r\right)=\chi \left(r\right),\;g\left(r\right)=\chi \left(r\right)\left(1+\delta \;\beta (r)\right),$$

so that the isotropy condition reduces to the ordinary differential equation

(35) $$\frac{\beta^{\prime }\left(r\right)}{\beta (r)}=-\frac{6}{3M+2\Lambda r^{3}-3r}.$$

In this framework, the energy–momentum tensor turns out to be exactly linear in δ, namely

(36) $$\begin{array}{cc} T_{\;0}^{0} & =-\Lambda +\left(-\Lambda +\frac{2}{r^{2}}-\frac{3(r-3M)}{r^{2}\left(3M+2\Lambda r^{3}-3r\right)}\right)\delta \;\beta \left(r\right), \end{array}$$

(37) $$\begin{array}{cc} T_{\;i}^{i} & =-\Lambda +\left(-\Lambda +\frac{1}{r^{2}}\right)\delta \;\beta \left(r\right), \end{array}$$

where β(r) is the solution of Equation (35), which is manifestly a linear differential equation. At this stage, then, we can take the parameter δ to be absorbed into the integration constant (denoted by *D*) emerging from Equation (35) by defining Υ:=Dδ. One is then left with a family of solutions parameterized by the single real constant Υ.

In general, the metric functions f(r) and g(r) obtained in this way do not necessarily share the same sign for all r>0, a condition that must be enforced in order to ensure a Lorentzian geometry, as discussed earlier.

Assuming M>0 and $\Lambda <1/(9M^{2})$, both f(r) and g(r) are positive in a neighborhood $\left(r_{+},r_{c}\right)$, where $r_{+}$ denotes the black hole horizon and $r_{c}$ the cosmological horizon. Furthermore, for Υ>0, one can show the existence of a radius $r_{0}\in \left(r_{+},r_{c}\right)$ at which curvature invariants diverge. In this case, the static geometry is well defined only in the interval $\left(r_{0},r_{c}\right)$ and exhibits a naked singularity as $r\to r_{0}$.

For Υ<0, the structure of the geometry is more involved, since g(r) vanishes at a radius $r_{\mathrm{thr}}\in \left(r_{+},r_{c}\right)$, although the metric remains regular at that location.

If M=0, one recovers our initial ansatz for f(r),

(38) $$f\left(r\right)=1-\frac{\Lambda }{3}r^{2},$$

which coincides with our solution in the limit ϵ=0.

This justifies our initial choice of f(r), made with the aim of finding g(r).

It can also be shown that, under the above assumptions on Λ and Υ, the metric is defined for all $r\in (r_{\mathrm{thr}},r_{c})$. By gluing two copies of such a solution at $r=r_{\mathrm{thr}}$, one can construct a wormhole geometry, as discussed in Ref. [37].

In summary, our metric is sufficiently general to reproduce both singular and regular solutions. When additional requirements are imposed, it also admits wormhole configurations.

## 4. Thermodynamics of Quasi-de Sitter Space

Below, we consider the special case of Equation (30) as a starting point, employing ϵ=0. The effects of deformation, induced by ϵ, have been discussed in Ref. [31] and appear to not affect the spacetime structure.

Since f≠g, the surface gravity, κ turns out to be

(39) $$\kappa =\frac{1}{2}\sqrt{|f^{\prime }\left(r_{h}\right)g^{\prime }\left(r_{h}\right)|},$$

and, so, the Hawking temperature becomes, in this case,

(40) $$\tilde{T}=\frac{\kappa }{2\pi }=\frac{1}{4\pi }\sqrt{|f^{\prime }\left(r_{h}\right)g^{\prime }\left(r_{h}\right)|},$$

where the tilde^˜^ indicates that this temperature is, in principle, different from *T*, which was previously introduced.

Accordingly, the specific heat becomes

(41) $$\tilde{C}=\tilde{T}\frac{d\tilde{S}}{d\tilde{T}},$$

under the assumption that $k_{0}$ is fully *independent of* Λ. This immediately yields

(42) $$\begin{array}{cc} \tilde{S} & =S, \end{array}$$

(43) $$\begin{array}{cc} \tilde{T} & =\frac{1}{2\pi }\sqrt{\left|k_{0}+\frac{1}{r_{h}^{2}}\right|}, \end{array}$$

(44) $$\begin{array}{cc} \tilde{C} & =-2\pi \left|k_{0}+\frac{1}{r_{h}^{2}}\right|r_{h}^{4}, \end{array}$$

which, in the limiting case, $k_{0}\to -\frac{2}{3}\Lambda$ reduces to $\tilde{T}\to T$, $\tilde{S}\to S$ and $\tilde{C}\to C$.

Alternatively, the specific heat is given by

(45) $$\tilde{C}=-\frac{8\pi^{3}T^{2}}{{\left(k_{0}-4\pi^{2}T^{2}\right)}^{2}},$$

where $k_{0}<0$ is an unavoidable condition, thereby forcing $\tilde{C}<0$.

A similarly intriguing outcome arises when expressing $\tilde{C}$ as

(46) $$\tilde{C}=-6\pi \left(\frac{\left|3k_{0}+\Lambda \right|}{\Lambda^{2}}\right),$$

**At first glance, there is no way to ensure thermodynamic stability.**

Nevertheless, if $k_{0}=k_{0}\left(r_{h}\right)$, then $\left[k_{0}\right]\equiv \left[\Lambda \right]\equiv {\left[L\right]}^{-2}$.

Hence, to guarantee that the metric *g* does not change its sign, i.e., that the Lorentzianity is preserved, we require

(47) $$\frac{1+k_{0}r^{2}}{1-\frac{2}{3}\Lambda r^{2}}>0,$$

since before the horizon, $r_{h}$, $1-\frac{\Lambda }{3}r^{2}>0$. Moreover, around r≃0, expanding to the second order gives

(48) $$g∼1+\left(k_{0}+\frac{\Lambda }{3}\right)r^{2}+\ldots ,$$

Therefore, without loss of generality, for a positive cosmological constant, the solution cannot be defined for all r>0 if our solution is not a de Sitter space.

Hence, for $k_{0}\leq -2\Lambda /3$, we expect to have $r\in ]0,1/\sqrt{-k_{0}}\left[\;\cup \;\right]\sqrt{3/(2\Lambda )},+\infty [$, corresponding to two patches. The first, $r\in ]0,1/\sqrt{-k_{0}}[$, is regular up to r=0, but with no horizon and, accordingly, no associated thermodynamics. The second patch has a singular boundary placed at $r=\sqrt{\frac{3}{2\Lambda }}$. In all other cases, the singularity is present and contained in the stationary region bounded by the horizon $r_{h}=\sqrt{\frac{3}{\Lambda }}$.

In addition, the case $-2\Lambda /3<k_{0}<-\Lambda /3$ provides a solution, defined within $r\in ]0,\sqrt{3/(2\Lambda )}\left[\;\cup \;\right]1/\sqrt{-k_{0}},+\infty [$.

The first patch is again regular, featuring a singular noncentral boundary at $r=\sqrt{3/(2\Lambda )}$. The other patch has an horizon but remains regular up to the boundary $r=1/\sqrt{-k_{0}}$.

Last but not least, for $-\Lambda /3\leq k_{0}<0$ appears similar to the above case, with the exception that the outer patch $r\in ]1/\sqrt{-k_{0}},+\infty [$ has no horizons. Finally, for $k_{0}\geq 0$, the outer patch disappears, leaving only the patch defined in the right neighborhood of r=0 given by $r\in ]0,\sqrt{3/(2\Lambda )}[\;,$ exists, which again contains a noncentral singularity.

All these considerations, together with the expansion of *g* around r≃0, suggest that the overall thermodynamics in the generalized de Sitter spacetime are not altered significantly, and the presence of $k_{0}$ appears as a shift in Λ.

Accordingly, as $k_{0}$ is negative, its primary effect is to reduce the specific heat without substantially modifying the overall thermodynamics, as stated above.

Last but not least, one can wonder how using Equation (29), instead of Equation (30) affects the specific heats. Since ϵ is very small, it does not influence the properties of the specific heat and, as has been shown in Ref. [31], its effect is not thermodynamic, so the overall results are largely unchanged and the specific heats remain negative.

### 4.1. Euclidean Derivation for the Temperature

The same procedure can be applied using the Euclidean formulation. Since our metric in Equation (30) satisfies f(r)≠g(r), our above treatment must be extended to this case, as we show below.

Near the horizon, we expand both the functions through

(49) $$f\left(r\right)\overset{r\to r_{h}}{\approx }f_{h}^{\prime }\left(r-r_{h}\right),\;g\left(r\right)\overset{r\to r_{h}}{\approx }g_{h}^{\prime }\left(r-r_{h}\right),$$

with $f^{\prime }\left(r_{h}\right)\equiv f_{h}^{\prime }$ and $g^{\prime }\left(r_{h}\right)\equiv g_{h}^{\prime }$, yielding, near the horizon,

(50) $$ds^{2}\approx -\frac{f_{h}^{\prime }\;z}{f_{0}}\;d\tau^{2}+\frac{1}{g_{h}^{\prime }z}\;dz^{2},$$

where we have used the fact that, for our metric, $f\left(r_{h}\right)=g\left(r_{h}\right)=0$. Moreover, we set dΩ=0 for simplicity and $r=r_{0}$ as a static point, where the observer measures proper time, so that $d\tau =\sqrt{f_{0}}\;dt,\;\mathrm{with}\;f_{0}=f\left(r_{0}\right)$.

Through a coordinate transformation, $dR\approx \frac{1}{\sqrt{g_{h}^{\prime }z}}\;dz$, we obtain

(51) $$ds^{2}\approx -\frac{f_{h}^{\prime }g_{h}^{\prime }}{4f_{0}}R^{2}\;d\tau^{2}+dR^{2}.$$

Following the same procedure used in Equation (19), we find here

(52) $${\tilde{T}}^{2}{\left(2\pi \right)}^{2}=\frac{f_{h}^{\prime }g_{h}^{\prime }}{4f_{0}}⇌\tilde{T}=\frac{\sqrt{\left|f_{h}^{\prime }g_{h}^{\prime }\right|}}{2\sqrt{f_{0}}\left(2\pi \right)},$$

This reduces to Equation (40) if $f_{0}=1$, certifying the viability of our analysis.

### 4.2. Comparative Remarks and Cosmological Implications

The quasi-de Sitter construction developed in this work naturally invites comparison with many modified gravity scenarios, including f(R) extensions, nonlinear electrodynamics (NLED) models, and semiclassical quantum-gravity corrections. There, the temperature and entropy of cosmological or black-hole horizons acquire model-dependent contributions. For instance, in NLED-based regular geometries [21], as well as in anti-de Sitter-inspired extensions and deformed black-bounce metrics, the thermodynamic quantities typically depend on additional curvature or on matter couplings that alter the effective gravitational sector.

In contrast, our solution relies on general relativity alone, as the metric modification enters exclusively through a geometrical integration constant $k_{0}$, without introducing higher-order curvature invariants or new propagating degrees of freedom.

Moreover, the case $k_{0}=0$ is, at least in principle, possible. This provides a clear benchmark by which to disentangle genuine nongeneral relativity thermodynamic effects from those induced simply by modifying the radial dependence of the metric functions.

Our analysis shows that, even when the metric is not written in Schwarzschild coordinates, the resulting thermodynamics remain formally identical to the de Sitter case once $k_{0}$ is re-expressed in terms of Λ.

We thus interpret the role of $k_{0}$ as an additional cosmological constant, which, however, does not influence the net thermodynamics.

This behavior may significantly differ across alternatives to general relativity [38,39], where additional couplings typically produce nonuniversal corrections to the horizon temperature or entropy. Although our treatment is analytical, the behavior of thermodynamic quantities can be complemented with a numerical exploration of the parameter space $\left(\Lambda ,k_{0}\right)$, as can be found in Ref. [31].

From a cosmological perspective, the invariance of the entropy-specific heat structure indicates that the quasi-de Sitter phase cannot mimic dark energy models featuring evolving effective vacuum energy, such as those driven by K-essence [40] or chiral-quintom fields [41].

Instead, the model represents a geometric deformation of de Sitter space that preserves the intrinsic thermodynamic signature of vacuum-dominated cosmologies.

This reinforces the interpretation that the observed thermodynamic instability is not an artifact of the deformation, but rather a robust feature of spherically symmetric general relativity backgrounds with a cosmological horizon.

### 4.3. Physical Interpretation of Negative Specific Heat and Thermodynamic Instability

The negative specific heat in our quasi-de Sitter configuration has important physical implications in cosmology, see e.g., [42].

More broadly, in gravitational systems, this property is commonly interpreted as signature for a self-gravitating instability: *an increase in internal energy leads to a decrease in temperature, preventing the construction of a canonical thermodynamic ensemble*.

This feature is quite common since, as stated above, it is even shared by the simplest Schwarzschild solution, as well as by more complicated spacetimes.

Within the present framework, the negative sign of the specific heat is not altered by $k_{0}$, which represents the parameter responsible for deviations from a de Sitter space.

This indicates that this instability is structurally tied to the combination of spherical symmetry and the existence of a cosmological horizon, although no theorem, so far, has mathematically demonstrated this.

The generalized de Sitter space modifies the local relation between (f,g) in de Sitter space but leaves the global thermodynamic behavior almost unchanged, suggesting that the instability is controlled primarily by the vacuum-energy term, namely Λ.

In quantum gravity and in approaches inspired by it, such as semiclassical corrections, the negative specific heat is sometimes interpreted as a signal of missing degrees of freedom in the coarse-grained thermodynamic description, which is clearly fully classical, as noted above in the context of the AdS/CFT correspondence.

Similarly, modified gravity models can map this property through the presence of additional curvature modes that redistribute energy nonlocally.

In contrast, our construction does not involve extra dynamical fields and the instability is thus genuinely geometric, originating from the global structure of the horizon itself.

These considerations reinforce the conclusion that resolving the thermodynamic instability of de Sitter-like spacetimes may require either

- breaking spherical symmetry in general relativity,
- including rotating or axisymmetric deformations, as prompted in Ref. [32], or
- extending general relativity by introducing additional degrees of freedom.

Moreover, for completeness, the negative behavior of specific heats can be associated with long-range interactions, which may be interpreted as a possible signature of nonlocal effects in gravity. More plausibly, however, the thermodynamic stability may be restored depending on the underlying underlying microphysics, a possibility that remains unclear in the literature.

## 5. Final Outlooks

In this work, we explored the thermodynamic properties of a quasi-de Sitter spacetime obtained by deforming the standard de Sitter space.

Specifically, we introduced two distinct procedures to derive the metric under consideration and provided two complementary interpretations of the resulting solution. We demonstrated that this geometry exhibits an asymptotically quintessence-like behavior, characterized by isotropic pressures and a nonconstant energy–momentum tensor and that it thereby differs substantially from the exact de Sitter configuration.

Moreover, we highlighted that a nonvanishing cosmological constant—or, equivalently, a vacuum energy contribution—persists in this scenario, with the metric admitting a finite radial domain wherein the Lorentzian signature is preserved.

A key feature of this construction is the emergence of an additional parameter, denoted $k_{0}$, whose thermodynamic implications we examined in detail. Even though $k_{0}$ is naively found by Einstein’s field equations, requiring isotropic pressure, its role appears *a priori* unspecified. Moreover, the modifications over the de Sitter phase do not depend on it alone, but also depend on a deformation on the shift function that also involves Λ.

Afterwards, we explicitly derived the entropy, Hawking temperature, and specific heat associated with this geometry, performing a comparative analysis with their de Sitter counterparts.

While the entropy remains unchanged, since the horizons are the same in both spacetimes under consideration, both the Hawking temperature and the specific heat acquire nontrivial corrections, parameterized by $k_{0}$. At first glance, these deviations appear to increase the complexity of the deformed metric; however, by interpreting $k_{0}$ as a parameter of the same dimensionality as the cosmological constant and imposing $k_{0}<0$ and $k_{0}<\frac{\Lambda }{3}$, we demonstrated that the thermodynamic quantities are slightly modified from those of de Sitter spacetime.

This reinforces the conclusion that the thermodynamic structure is intrinsically determined by the underlying geometric properties encoded in Λ alone, which remains formally identical between the quasi–de Sitter and de Sitter cases.

The issue of negative specific heat, along with its associated inconsistencies and indications of thermodynamic instability, remains here unresolved. Clearly, this depends on how f(r),g(r) and h(r) have been defined throughout our manuscript. Even though none of these quantities has been arbitrarily fixed, as stressed in the text, our metric deforms de Sitter space in a very precise and possibly nonunique manner. Alternatives may be explored in future efforts, although our findings appear consistent with the interpretation that these pathologies are inherently linked to spherical symmetry and to pure general relativity.

Several future directions naturally emerge, as summarized below.

(i) Turbulent and geometric instabilities. As discussed in Ref. [29], anti-de Sitter spacetime may exhibit weakly turbulent instabilities, where nonlinear mode coupling drives the system toward collapse. Given the similarity between our quasi-de Sitter model and de Sitter space, a full nonlinear stability analysis, combining perturbative techniques and numerical evolution, would be a useful future step.
(ii) Axisymmetric and rotating generalizations. Since the thermodynamic instability persists under spherical symmetry, a natural extension involves relaxing this symmetry and constructing axisymmetric solutions [32]. There, the role of the parameter $k_{0}$ would be re-explored, with the expectation that it would significantly differ from the present case.
(iii) Inclusion of matter sources and mass terms. Another promising direction concerns incorporating additional sources into the metric, such as scalar fields, anisotropic fluids or NLED. This prerogative will be investigated to understand how these sources influence the horizon thermodynamics.
(iv) Quantum and semiclassical corrections. The present work is fully classical; therefore, we intend to study semiclassical effects such as trace anomalies, one-loop corrections to the effective action, or nonperturbative quantum-gravity contributions to determine whether these effects can alter the sign of the specific heat.
(v) Euclidean quantum gravity and path-integral extensions. The Euclidean derivation presented here may be generalized by constructing the full Euclidean instanton associated with the quasi-de Sitter metric.
(vi) Implications for cosmology and vacuum-energy modeling. Since the asymptotic behavior of our solution resembles that of quintessence-like configurations, a systematic study of its cosmological embedding would be highly relevant, and, in particular, it would be worthwhile to explore whether the quasi-de Sitter phase may serve as an effective description of transitions between vacuum-dominated epochs, among other studies.

## Data Availability

The original contributions presented in this study are included in the article. Further inquiries can be directed to the corresponding author.
